# Investigation into the Effectiveness of an Herbal Combination (Angocin^®^ *Anti-Infekt N*) in the Therapy of Acute Bronchitis: A Retrospective Real-World Cohort Study

**DOI:** 10.3390/antibiotics13100982

**Published:** 2024-10-17

**Authors:** Nina Kassner, Meinolf Wonnemann, Yvonne Ziegler, Rainer Stange, Karel Kostev

**Affiliations:** 1 Repha GmbH Biologische Arzneimittel, 30855 Langenhagen, Germany; nina.kassner@repha.de (N.K.); meinolf.wonnemann@repha.de (M.W.); yvonne.ziegler@repha.de (Y.Z.); 2 Department for Internal and Integrative Medicine, Immanuel Hospital, 10117 Berlin, Germany; rainer.stange@immanuelalbertinen.de; 3 IQVIA Epidemiology, 60549 Frankfurt, Germany

**Keywords:** Angocin^®^ *Anti-Infekt N*, acute bronchitis, chronic bronchitis, antibiotics, sick leave, phytotherapy

## Abstract

Background: The goal of this study was to evaluate whether the medical recommendation of Angocin^®^ *Anti-Infekt N* (heretofore referenced as Angocin^®^) on the day of diagnosis of acute bronchitis is negatively associated with the recurrence of acute bronchitis diagnosis, antibiotic prescriptions, incidence of chronic bronchitis, and duration of sick leave. Methods: This study included patients in general practices in Germany with a first documented diagnosis of acute bronchitis between 2005 and 2022 (index date) and a prescription of Angocin^®^, thyme products, essential oils, mucolytics or antibiotics on the index date. The association between Angocin^®^ prescription and the risks of a relapse of acute bronchitis, development of chronic bronchitis, or subsequent antibiotic prescription were evaluated using Cox regression models. Univariable conditional logistic regression models were used to investigate the association between Angocin^®^ prescription and duration of sick leave. Results: After a 1:5 propensity score matching, 598 Angocin^®^ patients and 2990 patients in each of the four comparison cohorts were available for analysis. Angocin^®^ prescription was associated with significantly lower incidence of a renewed confirmed diagnosis of acute bronchitis as compared to essential oils (Hazard ratio (HR): 0.61; 95% Confidence Interval (CI): 0.46–0.80), thyme products (HR: 0.70; 95% CI: 0.53–0.91), mucolytics (HR: 0.65; 95% CI: 0.49–0.85) or antibiotics (HR: 0.64; 95% CI: 0.49–0.84). Also, there were significantly lower incidences of subsequent re-prescriptions of antibiotics when compared to mucolytics (HR: 0.73; 95% CI: 0.53–0.99) or antibiotics (HR: 0.53; 95% CI: 0.39–0.72) and a significantly lower risk of chronic bronchitis as compared to essential oils (HR: 0.60; 95% CI: 0.46–0.78), thyme products (HR: 0.53; 95% CI: 0.41–0.69), mucolytics (HR: 0.49; 95% CI: 0.38–0.63) or antibiotics (HR: 0.59; 95% CI: 0.45–0.76). Conclusions: Considering the limitations of the study, the results shed light on the sustaining effectiveness of Angocin^®^ prescription in the management of acute bronchitis and the associated outcomes when compared to several other treatments commonly used for this condition.

## 1. Introduction

Acute bronchitis is a temporary inflammation of the bronchial tubes, usually caused by a viral infection, though bacterial infections can also be responsible [1]. Typical symptoms range from cough, sputum production, rales on auscultation, chest pain on coughing to dyspnea [2]. Acute bronchitis usually lasts for a few days to a few weeks, but the cough can persist for several weeks after other symptoms have resolved. In most cases of acute bronchitis the symptoms resolve completely without any long-term complications. However, in some individuals, particularly those with certain risk factors, acute bronchitis can lead to chronic bronchitis [3]. Furthermore, in some cases, especially in individuals with impaired immune systems or underlying lung conditions, acute bronchitis can progress to pneumonia [4]. At present, the following antibiotics are most commonly prescribed for acute bronchitis in Germany: amoxicillin, azythromycin, and doxycyclin. Although antibiotics are generally not recommended unless a bacterial infection is present, antibiotic prescriptions are very frequent for acute bronchitis patients in primary care [5,6]. Treatment for acute bronchitis is usually supportive and focused on relieving symptoms. This may include over-the-counter medications for cough like ambroxol, acetylcysteine [7], or phytopharmaceutical products [8]. The efficacy and safety of herbal medicines in the treatment of acute bronchitis have been evaluated and confirmed in several clinical trials [9,10,11,12,13,14,15,16,17,18].The efficacy of herbal compounds is highly dependent on the plant, the extraction and the standardization methods used. The most prescribed phytopharmaceuticals by ATC classes were ivy products, essential oils like myrtol or cineole, thyme mono products and combinations, and others. Depending on the active ingredient, phytopharmaceuticals have antiviral, antibacterial, secretolytic, mucolytic, antiinflammatory, and / or immunomodulating properties. 

One of the phytopharmaceutical medications used for the therapy of acute bronchitis is Angocin^®^, a drug composed of nasturtium (*Tropaeolum majus* L.) and horseradish (*Armoracia rusticana* L.), licensed for over 50 years. Angocin^®^ is a medicinal product authorized in Germany, Austria and Switzerland, which fully complies with the requirements applicable in Europe with regard to efficacy and drug safety. It is not reimbursed by social security but rather by private health insurance for approximately 11% of the German population. The plant cultivation is performed according to Good Agricultural and Collection Practices (GACP). A detailed description of the manufacturing process has been reported previously [16]. Both plants are known to have anti-inflammatory and antimicrobial properties, among others, mediated by their content of isothiocyanates (also known as mustard oils). By combining the two plants, synergistic antibacterial effects were achieved *in vitro* [19,20]. 

Clinical studies have supported the effectiveness of Angocin^®^ in the management of acute bronchitis [16,21,22] as well as upper respiratory tract infections like sinusitis [21,22,23]. However, there is a lack of real-world evidence for the effectiveness of Angocin^®^ compared to standard antibiotic therapy as well as other phytopharmaceutical medications or synthetic drugs in patients with acute bronchitis. The goal of this study was to evaluate whether the medical recommendation of Angocin^®^ on the day of diagnosis of acute bronchitis is negatively associated with the recurrence of acute bronchitis diagnosis, antibiotic prescriptions, incidence of chronic bronchitis, and duration of the incapacity for work.

## 2. Methods

### 2.1. Data Source

This retrospective cohort study has the advantages over a prospectively planned clinical trial in that findings are obtained from the real use of a drug outside the narrow setting of a clinical trial, for example concerning study population, sample size and comorbidities. Furthermore, a comparison of several drug groups is possible. 

The study utilized the IQVIA^TM^ (formerly IMS Health) Disease Analyzer database, which serves as a comprehensive repository of electronic medical records containing patient demographics, diagnoses, and prescriptions. The data were collected from office-based physicians, including general practitioners (GPs), practicing in Germany. The Disease Analyzer database aggregates information from over 10 million patients, covering the period from 2005 to 2022. The selected practices represent diverse geographic distributions, spanning eight major regions across Germany. Data acquisition and procession strictly follow German data protection laws.

The sampling methods employed in Germany to select physicians’ practices are deemed suitable for establishing a representative database of both general and specialized practices, as demonstrated by Rathmann et al. in 2018 [24]. This widely recognized and utilized database has played a crucial role in numerous published studies focusing on acute respiratory tract infections, as evidenced by works such as those by Kern et al. [5] and Tanislav and Kostev [25].

### 2.2. Study Population

This study included patients in an outpatient care setting in Germany (general practitioners) with at least one diagnosis of acute bronchitis according to the “International Statistical Classification of Diseases and Related Health Problems” ICD code (ICD-10-GM: J20.-) from January 2005 to December 2022. Documentation of the three most common infections of bacterial etiology (J20.0, J20.1; J20.2) was rare, and therefore, not included in this analysis. Other types of infections were mostly coded as J20.8 or J20.9 (in total >99.4%).

Patients with a first documentation of acute bronchitis were categorized into one of five cohorts based on the prescription according to the “Anatomical Therapeutic Chemical” (ATC) classification system on the day of diagnosis:

- Angocin^®^ *Anti-Infekt N* (R05XP50);
- Thyme products (ATC: R05CP01 (thyme mono), R05CP51 (thyme combinations));
- Essential oils (ATC: R05CP59 (eucalyptus oil, combinations, e.g., myrtol), R05CA13 (cineole));
- Mucolytics (ATC: R05CB06 (ambroxol), R05CB01 (acetylcysteine), R05CB02 (bromhexine));
- Antibiotics (ATC: J01 (antibiotics for systemic use)).

The mentioned Anatomical Therapeutic Chemical code is a unique code assigned to a medicine according to the organ or system it works on and how it works. The classification system is maintained by the World Health Organization (WHO).

Patients with a prescription of study medication or other cough medications (ATC: R05) within 30 days prior to the index date, patients with a prescription of more than one study therapy on the index date, patients without documented information on age and sex and patients with a diagnosis of chronic bronchitis (ICD-10: J40-42) prior to or on index date were excluded for the analyses.

### 2.3. Propensity Score Matching

To reduce selection bias and impact of co-variables on the outcomes, we used a matched-pairs design. Among the methods given, propensity score matching seemed to be particularly effective in accounting for comorbidities, which are assumed to have a considerable impact on the outcome [26]. Patients with Angocin^®^ prescription were separately matched to each of the four study cohorts (1:5) using nearest neighbor propensity scores based on age, sex, health insurance status, Charlson Comorbidity Index (CCI) and diagnosis of asthma (ICD-10-GM-2024: J45) or chronic obstructive pulmonary disease (COPD) (ICD-10-GM-2024: J44) documented within 12 months prior to or on the index date. The CCI predicts the mortality for a patient who may have a range of comorbidities, such as heart disease, AIDS or cancer (considering a total of 17 categories [27]). In brief, a score of zero means that no comorbidities were found; the higher the score, the higher the predicted mortality rate [28,29]. The index was developed by Mary Charlson and colleagues. Each condition is assigned a score of 1, 2, 3 or 6, depending on the risk of dying associated with each one. Moreover, patients who are 50 years old or more obtained additional points [30]: 50–59 years old: +1, 60–69 years old: +2, 70–79 years old: +3, 80 years old or more: +4 points. Scores are summed to provide a total score to predict mortality. Conditions can be identified using the International Classification of Diseases (ICD). The Charlson index has been most commonly referred to by the comparative studies of comorbidity and multimorbidity measures [31]. Standardized mean difference (SMD) is the most frequently used measure to determine the balance of covariate distribution between treatment groups. In this study, we only allowed an SMD of less than 0.1 indicating that adequate covariate balance has been achieved [32].

### 2.4. Statistical Analyses

Patient data were collected for data analysis from the time of the first occurrence of acute bronchitis. This point in time was defined as the index date. The differences in proportions of patients with a renewed confirmed acute bronchitis diagnosis as well as antibiotic prescriptions within 1 to 365 days after diagnosis between the Angocin^®^ and any comparison cohort were evaluated using Kaplan–Meier curves. Univariable conditional Cox regression models were used to investigate the association between Angocin^®^ prescription and the probability of acute bronchitis recurrence or antibiotic prescription. Additionally, logistic regression models were calculated as proportional hazard assumptions were not met in some cases.

The difference in the cumulative incidence of chronic bronchitis up to three years after the index date was evaluated using Kaplan–Meier curves. The association between Angocin^®^ prescriptions and the risk of chronic bronchitis was estimated using conditional Cox regression models. 

The differences in percentages of patients with documented sick leave within one month following the prescription of Angocin^®^ or comparative medication were estimated in patients with at least one sick leave day. Furthermore, the proportions of patients with different sick leave durations (at least 4, resp. 7, resp. 14 days) were compared. Univariable conditional logistic regression models were used to investigate the association between Angocin^®^ prescription and the probability of sick leave (different durations). 

In all analyses, a *p*-value of <0.05 was considered statistically significant. Analyses were conducted using SAS Vers. 9.4 (SAS Institute, Cary, NC, USA).

## 3. Results

### 3.1. Baseline Characteristics of Study Patients

**Figure 1:**
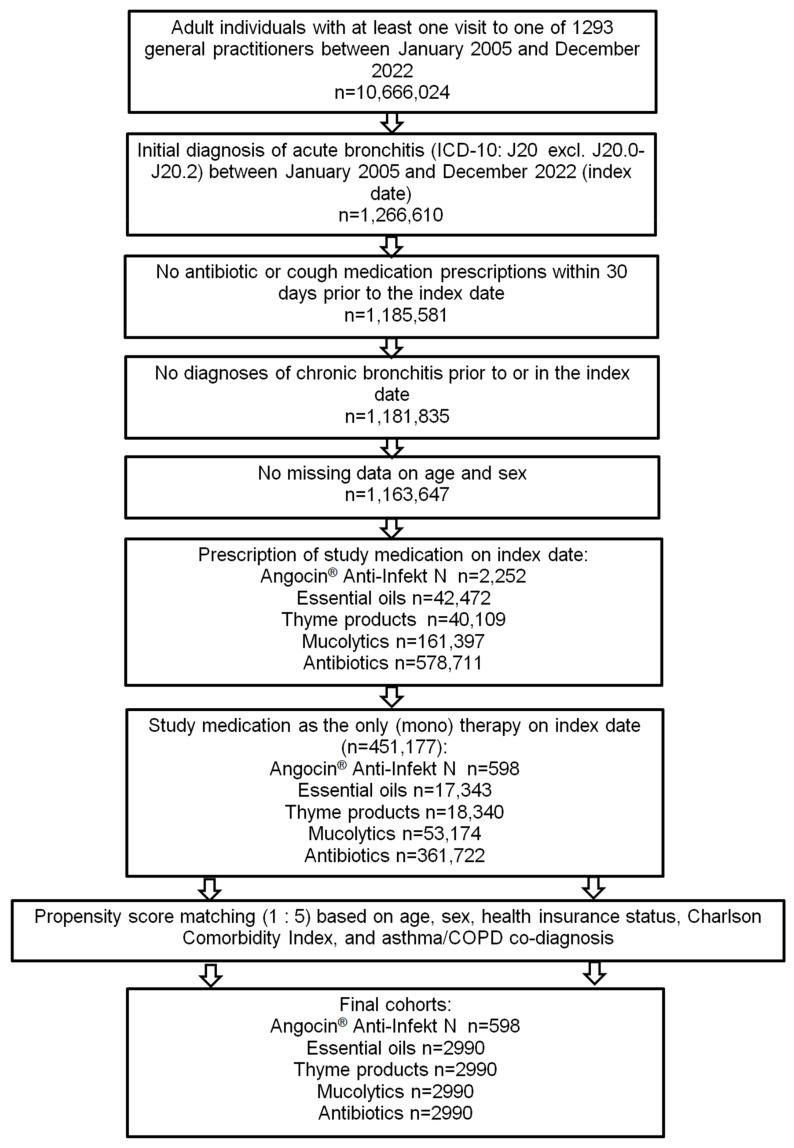
Selection of study patients.

Of 451,177 patients diagnosed with acute bronchitis and fulfilling all inclusion criteria, Angocin^®^ was prescribed to 598 patients, essential oils to 17,343, thyme products to 18,340, mucolytics to 53,174 and antibiotics to 361,722 patients. After 1:5 propensity score matching, 598 Angocin^®^ patients and 2990 patients in each of the four comparison cohorts were available for analysis (Figure 1). 

Table 1 shows the baseline characteristics of the study patients. After matching, no significant differences were observed between the study cohorts in terms of age (~42 years), sex (~59% female), private health insurance coverage (~10% private), diagnosis of asthma or COPD (~6%), or CCI (median 0).

### 3.2. Recurrence of Acute Bronchitis

**Figure 2:**
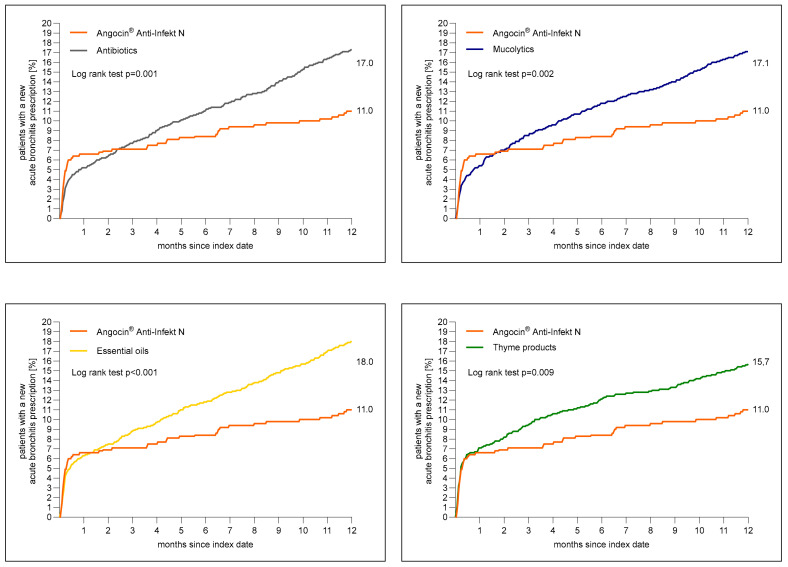
Cumulative incidence of a newly diagnosed bronchitis diagnosis 1 to 365 days (0 to 12 months) after index date in the Angocin^®^ cohort compared to the other therapies (Kaplan–Meier curves).

Figure 2 shows the cumulative incidence of newly diagnosed acute bronchitis. A total of 11.0% of patients with Angocin^®^ prescription and >16% of patients in each further cohort received a diagnosis of acute bronchitis 1 to 365 days after the index date. 

The results of the Cox regression and logistic regression models are displayed in Table 2. Angocin^®^ prescription was associated with significantly lower incidence of a renewed confirmed diagnosis of acute bronchitis as compared to essential oils (Hazard ratio (HR): 0.61; 95% Confidence interval (CI): 0.46–0.80), thyme products (HR: 0.70; 95% CI: 0.53–0.91), mucolytics (HR: 0.65; 95% CI: 0.49–0.85) and antibiotics (HR: 0.64; 95% CI: 0.49–0.84) within 1–365 days after index date. Logistic regression analysis confirmed the associations found in Cox regression analysis (Table 2).

### 3.3. Antibiotic Prescriptions after the Index Date

**Figure 3:**
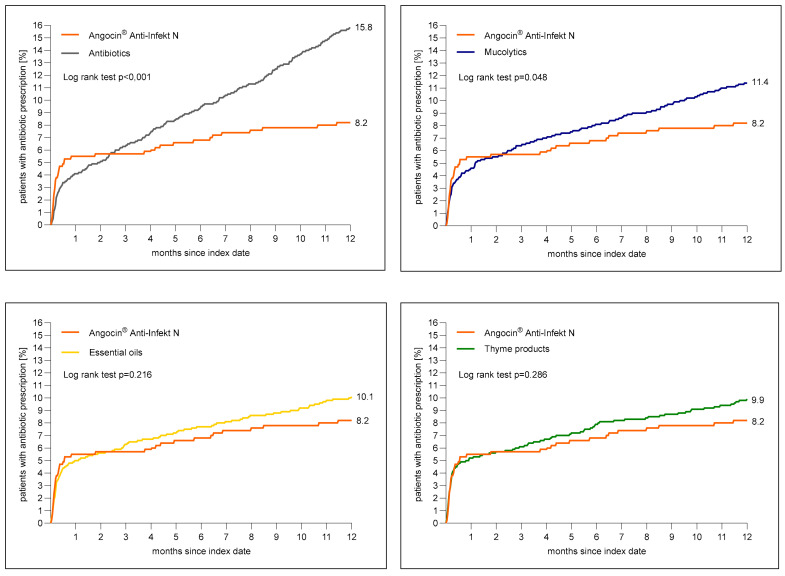
Cumulative incidence of antibiotic prescription within 1 to 365 days (0–12 months) after index date in the Angocin^®^ cohort compared to the other therapies (Kaplan–Meier curves).

Figure 3 shows the cumulative incidence of an antibiotic prescription within 1 to 365 days after the index date. The cumulative incidence was at lowest in the Angocin^®^ cohort (8.2%) and at highest in the antibiotic cohort (15.8%).

The results of the Cox regression and logistic regression models are displayed in Table 3. Angocin^®^ prescription was associated with a significantly lower incidence of an antibiotic prescription as compared to mucolytics (HR: 0.73; 95% CI: 0.53–0.99) and antibiotics (HR: 0.53; 95% CI: 0.39–0.72) within 1–365 days after the index date. No significant associations were observed for Angocin^®^ compared to essential oils (HR: 0.82; 95% CI: 0.0–1.12) and thyme products (HR: 0.84; 95% CI: 0.61–1.16) (Table 3).

### 3.4. Incidence of Chronic Bronchitis

The cumulative incidence of chronic bronchitis was lowest in the Angocin^®^ cohort (9.2%) and between 13.1% to 15.8% in other cohorts. In Cox regression models, Angocin^®^ was significantly associated with a lower risk of chronic bronchitis as compared to essential oils (HR: 0.60; 95% CI: 0.46–0.78), thyme products (HR: 0.53; 95% CI: 0.41–0.69), mucolytics (HR: 0.49; 95% CI: 0.38–0.63), or antibiotics (HR: 0.59; 95% CI: 0.45–0.76) within three years after index date (Table 4).

### 3.5. Sick Leave Associated with Acute Bronchitis

**Figure 4:**
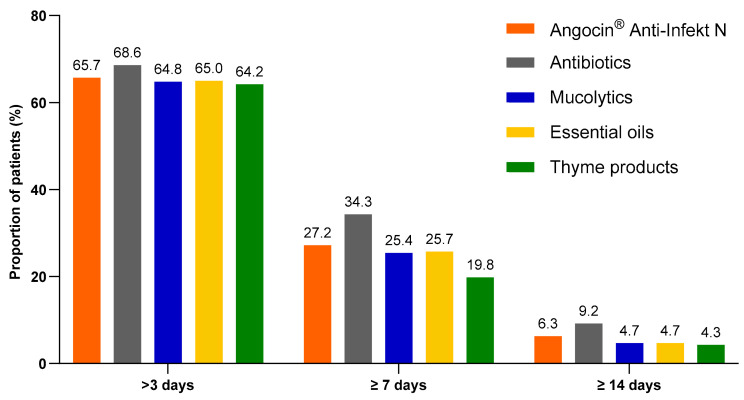
Proportion of patients with a sick leave duration of >3, ≥7 and ≥14 days.

Among patients with at least one day of sick leave included in the analysis, the proportion of patients with antibiotics who were on sick leave for more than 3, at least 7 and at least 14 days was the highest. However, the proportion of Angocin^®^ patients with defined sick leave durations was some higher than in other cohorts excluding antibiotics (Figure 4). 

The results of the multivariate logistic regression model are displayed in Table 5. As only 268 Angocin^®^ patients were available for sick leave analysis, the results of this analysis should be treated with caution. In most cases, no significant association was observed between Angocin^®^ and sick leave duration. Only compared to antibiotics Angocin^®^ was associated with lower odds of sick leave ≥7 days (Odds ratio (OR): 0.72; 95% CI: 0.52–0.99). At the same time, compared to thyme products, Angocin^®^ was associated with higher odds of sick leave ≥7 days (OR: 1.52; 95% CI: 1.08–2.13).

## 4. Discussion

The present retrospective study using propensity score matching design and systematically retrieved data from clinical practice shows that the use of Angocin^®^ as first-line treatment for acute bronchitis is associated with decreased risks for relapse as well as the development of chronic bronchitis when compared to other commonly prescribed medications. Furthermore, it is also associated with a reduced risk of antibiotic prescriptions when compared to mucolytics or antibiotics as initial therapy.

Comparison with antibiotics could be critically questioned as it could be assumed that patients who received antibiotics on the day of diagnosis were more severely ill than those who were treated with phytodrugs like Angocin^®^ and other over-the-counter medication. However, this assumption can be contradicted. First, Ehrenberg and colleagues investigated factors associated with a prescription of phytopharmaceuticals in an outpatient setting in Germany, showing that a practice preference for phyto was associated with six-fold increased odds of phytopharmaceutical prescription, independent of diagnosis and patient characteristics [33]. Furthermore, it is assumed that around 10% of cases of acute bronchitis are caused by bacteria, especially in patients with underlying health conditions [34]. 

The study also showed that explicit coding of common bacterial etiologies by specific test diagnoses is rare (0.2%), and diagnoses of viral or undefined infections are very frequent. Since the choice of therapy has to be made quickly, nowadays, available laboratory tests for distinguishing between infections of viral or bacterial origin are rarely performed in clinical practice due to the delayed results after several days and the associated costs. A total of 96.1% of the included diagnosis was “J20.9 Acute bronchitis, unspecified”. However, in our study, prior to matching, 80.2% received antibiotics on the day of diagnosis. This suggests that many of the patients who received antibiotics may not have a proven bacterial infection. This is because doctors often have to judge according to other insecure diagnostic criteria such as sputum color, fever and general impression, resulting in a certain degree of misdiagnosis. In cases of viral etiology, antibiotics cannot work. Moreover, antibiotic administration can weaken the protective immune memory, and patients can become increasingly susceptible to new viral infections [35]. This high proportion of antibiotic use in acute bronchitis was previously discussed by Kern and Kostev [5]. However, in cases of bacterial infections, good effectiveness of Angocin^®^ can also be expected due to strong antibacterial effects against relevant pathogens *in vitro*. Published research indicated that *Tropaeolum majus* and *Armoracia rusticana* contain the active compounds, the isothiocyanates, with potent antibacterial effects also against multiresistant strains suggesting potential applications of these plants in developing new antimicrobial treatments [19,20,36,37].

In this study, Angocin^®^ was also compared to mucolytics. Looking at the prescription data, mucolytics are very often used in the investigated indication. Indeed, some evidence gleaned from clinical and observational trials, along with practical, real-world experience, indicates that mucolytics may be beneficial in treating respiratory tract infections such as bronchitis [38]. Unfortunately, there are currently no large epidemiological studies comparing these drugs with phytopharmaceuticals. The present study provides new evidence supporting the efficacy of Angocin^®^ in the therapy of both acute and chronic bronchitis directly compared to mucolytics. The observed long-term effect is particularly relevant as it was also observed in the comparison of Angocin^®^ with other study cohorts. The impact of the ITC combination in Angocin^®^ on different stages of bacterial biofilm formation as well as on metabolic activity in mature biofilms could be essential for this observation [36]. The ability to form biofilms is a pathogenic virulence factor, which is thought to play a role in several relevant aspects of infection pathology, e.g., relapses, chronification and also the development of antibiotic resistance.

Interestingly, the association between Angocin^®^ compared to essential oils and thyme products with reduced risk of new bronchitis diagnoses, both acute and chronic, is new. Yet, the effectiveness of cineole compared to placebo was investigated only in a clinical trial [13] and neither results of real-world studies are available nor comparisons with other phytopharmaceuticals in the therapy of acute bronchitis. The anti-inflammatory effects of thyme products were demonstrated in vitro and in vivo studies and clinical trials compared to placebo [9,39] but not in real-world studies. Similarly, anti-inflammatory effects by blocking lipoxygenase and cyclooxygenase pathways could be reported for nasturtium and horseradish and for the combination of both [40,41,42]. 

The symptoms of acute bronchitis caused by a viral or, less often, bacterial infection were characterized by an inflamed epithelium of the bronchial tubes and strong mucous secretion. The antiviral properties of Angocin^®^ on typical enveloped and non-enveloped viral strains are currently extensively investigated. So far, antiviral properties have been shown for similar main components of the isothiocyanate family, like L-sulforophane, against the quite common respiratory syncytial virus (RSV) [43] and, most recently, against the SARS-Cov2 virus [44]. Also, the proven antibacterial effects of Angocin^®^ against a broad spectrum of clinically relevant pathogens, including multidrug-resistant strains [19,20] and inhibition of biofilm formation, could also play a role in the treatment and prevention of acute bronchitis. Furthermore, it could be assumed that the treatment effects described above might be due to an activation or upregulation of parts of the innate immune system. It is already known that several plant extracts can upregulate certain defensins, which serve as a natural defense against microbial pathogens, especially bacteria, fungi and toxins [45,46]. 

In a nasturtium consumption study, it was shown that the ingestion of active, ITC-releasing nasturtium by healthy volunteers leads to a significant increase in human beta-defensin-1 (hBD-1) in urine and breath condensate [47]. Human beta-defensin-1, as part of the innate immune response, is an antimicrobial peptide and is predominantly produced on epithelial cells of the respiratory and urinary tract for the elimination of viruses, bacteria and fungi. Knockout mice (Defb1-knock out), which no longer produce homologous murine defensin 1 (DEFb1), showed delayed bacterial clearance of the lung and increased staphylococcal infection in the bladder. Yet, infections with different, respiratory viruses or bacteria also lead to an upregulation in human beta defensins and effects on the acute humoral immune response cannot fully explain the long-term effects seen in this analysis.

It is known, from an earlier study, that the long-term intake of Angocin^®^ can reduce the rate of further respiratory tract infections. In a randomized, prospective, placebo-controlled, double-blind 12-week study, 344 subjects were divided into 3 groups. They either took 3×2 tablets of Angocin^®^ (group 1), 2×2 tablets of Angocin^®^ plus 1×2 placebo tablets (group 2) or 3×2 placebo tablets (placebo group) daily for 12 weeks. The primary study endpoint was the frequency of cold episodes during the 12-week treatment period. A clinically relevant and statistically significant difference in favor of the high dosage versus placebo (*p* = 0.0171) could be demonstrated: the infection rate was 13.3% in the first group, 18.4% in group 2, and 25.6% in the placebo group. Almost 50% fewer colds occurred in the verum group with 3×2 tablets than in the placebo group. Moreover, the phytotherapeutic agent proved to be safe and was well tolerated by 94% of the test subjects. [48]. This outcome is in line with the results of this retrospective study. 

## 5. Limitations

The present study is subject to a number of relevant studies and database limitations. These apply to many studies of this type. While retrospective studies have the advantage of tracking characteristics over a long period of time with large sample sizes, data sets cannot comprehensively include all details about patients. In our analysis, we included all factors available about patients and their diseases, describing limitations in the discussion.

The database does not include data on the use of herbal medicines and other OTC drugs purchased without a prescription. Furthermore, the database does not include data on symptoms in the case of such diagnoses whereby symptoms can be responsible for the choice of defined therapy. 

No data are available on socioeconomic status and lifestyle-related risk factors, whereby smoking behavior, in particular, would be relevant in the present analysis. Moreover, all diagnoses were assessed based on ICD codes entered by GPs, and these codes do not necessarily allow a distinction to be made between viral and bacterial infections on the day of prescription as well as between different severity stages of diseases. Another limiting point of this data analysis is the lack of knowledge about a possible general prescribing behavior of physicians, i.e., whether there may be a preference for a non-antibiotic or antibiotic treatment or of the factors that trigger the choice of the therapy in the case of reinfection. Moreover, the analysis cannot reflect the opinions or certain wishes of an affected patient. ICD-10 chapter J20.-, with all decimals, is clearly orientated towards etiology and does not offer any hint of severity, which thus could not be imputed in our analysis. Since doctors’ prescribing attitudes were not known, it cannot be estimated to what extent severity had a role in their method of prescribing.

Finally, propensity score matching is a way to control for confounding variables and to mimic the effect of randomization with retrospective data. In this way, the study can ensure that differences in the results between the groups can be attributed to the treatment. Compared to traditional exact or hard matching, propensity score matching offers greater freedom in terms of efficiency and control of bias in studies, in which many variables have to be taken into account. Of course, this method is inherently limited because one can only control for a few relevant covariates; stratifying on too many covariates, however, will result in groups that are too sparse to estimate effects. Matching criteria were chosen to allow for robustness and balance as well as causal plausibility and minimal confounder bias, while the resulting sample sizes still allowed valid results. This was especially important as the Angocin^®^ cohort strongly differed from other cohorts in health insurance status—the majority of Angocin patients are privately insured—which could entail a very large bias. Moreover, this status could be linked to the social status of patients since Angocin^®^ likewise is not reimbursed by the social security system in Germany. Incidentally, we strictly adhered to the best practice recommendations of Chen J.W. et al. [49]. 

An alternative approach would have been a multivariable regression analysis including all patients with adjustment of co-variates. In particular, robustness was checked in a sensitivity analysis without matching but adjusted to age, sex, health insurance status, CCI and pre-existing diseases. The overall results were not significantly different. The choice of the best methodology is a general matter of opinion. 

## 6. Conclusions

The study results shed light on the effectiveness of Angocin^®^ prescription in the ma-nagement of acute bronchitis and its associated outcomes compared to several other treatments commonly used for this condition. Considering all the limitations of these real-world data, the findings of our analyses suggest that Angocin^®^ is negatively associated with a risk of recurrent acute bronchitis and chronic bronchitis compared to other commonly prescribed medications. Furthermore, Angocin^®^ might be associated with a reduced need for antibiotic prescriptions compared to mucolytics and antibiotics themselves. The findings should definitely be confirmed in further examinations. Given the growing concern over antibiotic resistance and the need to minimize unnecessary antibiotic use, Angocin^®^ might be a possible alternative.

Angocin^®^ has already been proven as an effective and well-tolerated therapy for acute bronchitis [9]. However, further research, particularly prospective long-term studies in comparison to antibiotics, and exploring the mechanisms underlying the observed associations, would be meaningful.

## Figures and Tables

**Table 1:** Basic characteristics of study patients.

Variable	Patients with Angocin^®^ Prescription	Patients with Essential Oils Prescription	Patients with Thyme Products Prescription	Patients with Mucolytics Prescription	Patients with Antibiotic Prescription	*p*-Value
N	598	2990	2990	2990	2990	
Mean age (SD)	41.7 (17.8)	41.9 (17.5)	41.5 (18.4)	41.8 (18.1)	41.8 (17.9)	0.881
<18 years (N, %)	37 (6.2)	163 (5.5)	214 (7.2)	194 (6.5)	182 (6.1)	0.838
18–30 years (N, %)	147 (24.6)	742 (24.8)	751 (25.1)	732 (24.5)	738 (24.7)	
31–45 years (N, %)	170 (28.4)	852 (28.5)	810 (27.1)	838 (28.0)	849 (28.4)	
46–65 years (N, %)	186 (31.1)	938 (31.4)	904 (30.2)	915 (30.6)	925 (30.9)	
>65 years (N, %)	58 (9.7)	295 (9.9)	311 (10.4)	311 (10.4)	296 (9.9)	
Sex: female (N, %)	354 (59.2)	1765 (59.0)	1775 (59.4)	1743 (58.3)	1787 (59.8)	0.836
Private health insurance coverage (N, %)	62 (10.4)	295 (9.9)	291 (9.7)	275 (9.2)	300 (10.0)	0.806
CCI (median, interquartile range)	0 (1)	0 (1)	0 (1)	0 (1)	0 (1)	0.504
Asthma/COPD (N, %)	36 (6.0)	148 (5.0)	168 (5.6)	171 (5.7)	182 (6.1)	0.406

**Table 2:** Association between Angocin^®^ prescription and renewed confirmed diagnosis of acute bronchitis 1 to 365 days after the index date (Cox and logistic regression models).

	Cox Regression		Logistic Regression	
	Hazard Ratio (95% CI)	*p*-Value	Odds Ratio (95% CI)	*p*-Value
Angocin^®^ vs. essential oils	0.61 (0.46–0.80)	<0.001	0.62 (0.46–0.82)	<0.001
Angocin^®^ vs. thyme products	0.70 (0.53–0.91)	0.009	0.70 (0.53–0.94)	0.015
Angocin^®^ vs. mucolytics	0.65 (0.49–0.85)	0.002	0.66 (0.50–0.88)	0.004
Angocin^®^ vs. antibiotics	0.64 (0.49–0.84)	0.001	0.65 (0.49–0.86)	0.003

**Table 3:** Association between Angocin^®^ prescription and antibiotic prescription 1 to 365 days after the index date (Cox and logistic regression models).

	Cox Regression		Logistic Regression	
	Hazard Ratio (95% CI)	*p*-Value	Odds Ratio (95% CI)	*p*-Value
Angocin^®^ vs. essential oils	0.82 (0.60–1.12)	0.216	0.85 (0.62–1.19)	0.349
Angocin^®^ vs. thyme products	0.84 (0.61–1.16)	0.286	0.86 (0.62–1.19)	0.362
Angocin^®^ vs. mucolytics	0.73 (0.53–0.99)	0.049	0.76 (0.55–1.05)	0.096
Angocin^®^ vs. antibiotics	0.53 (0.39–0.72)	<0.001	0.52 (0.38–0.72)	<0.001

**Table 4:** Association between Angocin^®^ prescription and the risk of chronic bronchitis (Cox regression models).

	Hazard Ratio (95% CI)	*p*-Value
Angocin^®^ vs. essential oils	0.60 (0.46–0.78)	<0.001
Angocin^®^ vs. thyme products	0.53 (0.41–0.69)	<0.001
Angocin^®^ vs. mucolytics	0.49 (0.38–0.63)	<0.001
Angocin^®^ vs. antibiotics	0.59 (0.45–0.76)	<0.001

**Table 5:** Association between Angocin^®^ prescription and probability of sick leave of >3, ≥7 and ≥14 days [Angocin^®^ versus other therapies]. Significant *p*-values are highlighted in bold.

	>3 Days		≥7 Days		≥14 Days	
Sick Leave Duration	Odds Ratio (95% CI)	*p*-Value	Odds Ratio (95% CI)	*p*-Value	Odds Ratio (95% CI)	*p*-Value
Angocin^®^ vs. essential oils	1.03 (0.77–1.38)	0.846	1.08 (0.79–1.49)	0.616	1.36 (0.75–2.48)	0.314
Angocin^®^ vs. thyme products	1.07 (0.79–1.45)	0.669	1.52 (1.08–2.13)	**0.015**	1.52 (0.81–2.87)	0.196
Angocin^®^ vs. mucolytics	1.04 (0.77–1.41)	0.805	1.10 (0.79–1.53)	0.570	1.37 (0.73–2.55)	0.329
Angocin^®^ vs. antibiotics	0.88 (0.64–1.20)	0.412	0.72 (0.52–0.99)	**0.044**	0.67 (0.38–1.18)	0.164

## Data Availability

The data presented in this study are available upon request from the corresponding author.
